# Differential methylation of linoleic acid pathway genes is associated with PTSD symptoms – a longitudinal study with Burundian soldiers returning from a war zone

**DOI:** 10.1038/s41398-024-02757-7

**Published:** 2024-01-18

**Authors:** Anselm Crombach, Anja C. Rukundo-Zeller, Vanja Vukojevic, Corina Nandi, Manassé Bambonye, Dominique J.-F. de Quervain, Andreas Papassotiropoulos, Thomas Elbert

**Affiliations:** 1 Department of Psychology, Clinical Child and Adolescent Psychology and Psychotherapy, Saarland University,, Saarbrücken, Germany; 2 Department of Psychology, Université Lumière de Bujumbura, Bujumbura, Burundi; 3 Department of Psychology, Clinical Psychology and Neuropsychology, University of Konstanz, Konstanz, Germany; 4 Department of Biomedicine, Research Cluster Molecular and Cognitive Neurosciences, University of Basel, Basel, Switzerland; 5 University Psychiatric Clinics, University of Basel, Basel, Switzerland

**Keywords:** Genetics, Psychology

## Abstract

Soldiers may be exposed to traumatic stress during combat deployment and thus are at risk for developing posttraumatic stress disorder (PTSD). Genetic and epigenetic evidence suggests that PTSD is linked to forming stress-related memories. In the current study, we investigated post-deployment associations of PTSD symptoms with differential DNA methylation in a sample of Burundian soldiers returning from the African Union Mission in Somalia’s war zone. We used a matched longitudinal study design to explore epigenetic changes associated with PTSD symptoms in *N* = 191 participants. PTSD symptoms and saliva samples were collected at 1–3 (t1) and 9–14 months (t2) after the return of the soldiers to their home base. Individuals with either worsening or improving PTSD symptoms were matched for age, stressful, traumatic and self-perpetrated events prior to the post-assessment, traumatic and violent experiences between the post- and the follow-up assessment, and violence experienced during childhood. A mixed model analysis was conducted to identify top nominally significantly differentially methylated genes, which were then used to perform a gene enrichment analysis. The linoleic acid metabolism pathway was significantly associated with post-deployment PTSD symptoms, after accounting for multiple comparisons. Linoleic acid has been linked to memory and immune related processes in previous research. Our findings suggest that differential methylation of linoleic acid pathway genes is associated with PTSD and thus may merit closer inspection as a possible mediator of resilience.

## Introduction

During war deployments soldiers are frequently exposed to traumatic stress [1, 2]. As the likelihood of developing posttraumatic stress disorder (PTSD) increases with each additional traumatic experience via a building block effect [3–5], soldiers face an elevated risk of developing PTSD with increasing involvement in violence [1, 2, 6, 7]. Research on neurobiological correlates, such as associated memory processes, suggests that the experience of multiple traumatic events leads to the build-up of a fear network [8, 9]. These networks are generated by the strong physiological arousal during trauma exposure, which reinforces an associative memory representation between trauma-related perceptual cues, cognitions, emotions, and physiological reactions. With an increasing number of traumatic events, the connection between the contextual information of specific events, i.e., factual knowledge about where and when the event happened, and the trauma responses to those events become more and more disconnected, causing an overgeneralisation of this fear network. This disconnection between the place and time of a specific event, and the blurring of trauma-related cognitive, emotional and physiological reactions, causes the fear network to be easily triggered, and subsequently prompt PTSD symptoms such as flashbacks and intrusions [10].

The importance of the interplay between stress and memory processes for the development of trauma-related symptoms is further corroborated by studies linking PTSD to epigenetic modifications of stress systems such as the hypothalamus–pituitary–adrenal axis [11], associated memory related processes [12], and changes within the immune system [13, 14], amongst others. NOTCH pathways, which are associated, for example, with inflammatory signalling and fear memory consolidation, have been linked to lifetime PTSD risk in a Ugandan sample, suggesting a possible mediator role of NOTCH for PTSD risk after trauma [15].

One of the most frequently studied epigenetic markers in humans is DNA methylation (DNAm). This can affect the transcription of DNA without changing the underlying genetic sequence. Several epigenome-wide association studies (EWAS) investigated DNAm changes in relation to PTSD in longitudinal designs with soldiers, assessing changes in methylation patterns across the genome pre- and post-deployment. In 429 male soldiers from the United States and the Netherlands, Katrinli et al. [16] identified cytosine-phosphate-guanine (CpG) sites near the genes *F2R, CNPY2, BAIAP2L1*, and *TBXAS1* to be associated with risk of PTSD within military personnel prior to and following deployment. These genes might affect PTSD symptoms via modulating the immune response (F2R, TBXAS1), performance of hypothalamic cells (CNPY2), central nervous system development (CNPY2, BAIAP2L1), and by altering the striatal dopamine level (TBXAS1). Moreover, amongst 15 differentially methylated regions associated with PTSD symptoms, several were related to genes important for immune system functioning.

Rutten et al. [17] found that DNAm levels decreased in 93 Dutch soldiers during deployment to Afghanistan in regions located on the *ZFP57*, *RNF39* and *HIST1H2APS2* genes. These changes in methylation levels were associated with increased PTSD symptoms after their deployment. *ZFP57* is involved in the genetic imprinting of stem cells, whereas *RNF39* has been linked to an increased risk of several psychiatric disorders, as well as in synaptic plasticity and long-term potentiation. Another longitudinal meta-analysis investigating changes in methylation levels pre- and post-deployment in three cohorts of United States and Dutch soldiers (total *N* = 266) revealed that three CpG sites, cg05656210 (intergenic), cg12169700 located on the *MAD1L1* gene, and cg20756026 on the *HEXDC* gene, were associated with PTSD [18]. According to the authors, these findings indicate associations between PTSD and epigenetic factors associated with learning and memory processes, the immune system, and stable DNA replication in mitosis.

In summary, previous longitudinal EWAS assessing PTSD and DNAm in soldiers pre- and post-combat missions have found epigenetic loci related to the immune system, synaptic plasticity, and long-term potentiation, imprinting of stem cells, apoptosis, and cell communication. The existing longitudinal studies referred to samples of mostly White US and Dutch soldiers and assessed changes in methylation patterns associated with PTSD symptoms throughout combat missions. In contrast, our study focuses on identifying methylation patterns in the aftermath of returning from a one-year military deployment, which might differentiate between soldiers whose PTSD symptoms improve or deteriorate. To this end, we assessed Burundian soldiers who had been deployed in the African Union Mission in Somalia (AMISOM) directly after their return from the war zone to their home base, and one year later. In the current study we aimed to identify epigenetic methylation patterns associated with improving or deteriorating PTSD symptoms in a group of soldiers after combat experience.

## Methods

### Sample

The sample consisted of 191 active duty male Burundian soldiers (*M*_*age*_ = 34.54, *SD*_*age*_ = 4.59, range = 22–53 years). The data was collected as part of a longitudinal study assessing mental health issues in Burundian soldiers before (t0), 1–3 months (t1) and 9–14 months (t2) after their return from their deployment within the AMISOM in the years 2013 and early 2014. The present study used data from the post-deployment (t1; 1–3 months after return to Burundi) and 9–14 months follow-up assessment post-deployment (t2). Given that we were interested in the differential methylation that could be associated with post-deployment change of PTSD symptoms, for the current study we have utilised data from t1 and t2. The chosen participants were selected and matched in two groups: (1.) a group of soldiers with deteriorating PTSD symptoms between post-assessment and follow-up assessment, and (2.) a group of soldiers with improving PTSD symptoms over time. For more information on the entire sample, see Nandi et al. [19–21]. Table 1 provides a descriptive overview of the sample.

**Table 1:** Descriptive statistics and group comparisons of the samples.

Variable	Total	Improving (*n* = 100)	Deteriorating (*n* = 91)	*t* test
Years of school, *M* (*SD*) [range]	6.07 (2.13) [0–20]	6.24 (2.47) [0–20]	5.89 (1.69) [0–10]	*t*_175.96_ = 1.15
t1				
Age, *M* (*SD)* [range]	34.45 (4.62) [22–53]	34.54 (4.65) [25–45]	34.34 (4.60) [22–53]	*t*_187.69_ = 0.30
PTSD sum score, *M* (*SD)* [range]	4.55 (5.38) [0–23]	6.97 (5.14) [1–23]	1.88 (4.29) [0–23]	*t*_187.64_ = 7.46***
Traumatic event types until t1, *M* (*SD)* [range]	24.21 (3.26) [5–38]	24.53 (6.42) [5–38]	23.86 (6.47) [8–37]	*t*_187.05_ = 0.72
t2				
PTSD sum score, *M* (*SD)* [range]	4.24 (5.99) [0–29]	1.23 (2.28) [0–12]	7.54 (6.99) [1–29]	*t*_107.33_ = −8.22***
Traumatic event types t1 to t2, *M* (*SD)* [range]	1.70 (1.74) [0–9]	1.64 (1.61) [0–9]	1.77 (1.89) [0–9]	*t*_177.59_ = −0.51
Childhood maltreatment, *M* (*SD)* [range]	10.34 (4.30) [1–24]	10.21 (4.07) [1–18]	10.47 (4.55) [2–24]	*t*_181.27_ = −0.42

*M* mean, *SD* standard deviation, asterisks indicate statistical significance: ***p < 0.001.

### Study procedure

At each investigation time-point, clinical interviews were conducted, and saliva samples collected. For more details regarding study design see Nandi et al. [19–21] and Moran et al. [22]. The clinical assessments were conducted using standardised clinical interviews. Trained mental health experts from Burundi and Germany (with the help of interpreters) conducted the interviews in Kirundi. The ethics committees of the University Lumière de Bujumbura, of the University of Konstanz, and the European Research Council approved the study procedures.

**Fig. 1:**
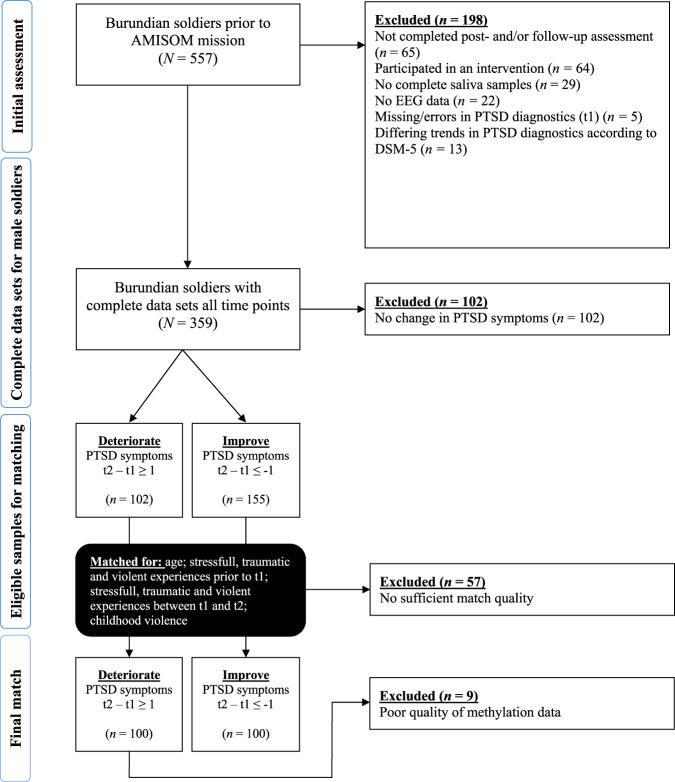
Flow-chart documenting the selection and matching process. The flow-chart shows the reasons for excluding participants, and the matching procedure assigning the participants into groups with either ameliorating or deteriorating PTSD symptoms between t1 and t2.

To investigate the association between DNAm and PTSD symptom development over time, we identified 200 individuals with contrasting PTSD symptom trajectories between post- and follow-up assessment. By focusing on the extremes in our data set and subsequent matching, we increased the power of our analysis. As indicated in the flow-chart (Fig. 1), we excluded in a first step all individuals with incomplete data sets. Furthermore, we excluded 64 soldiers who participated in a preventive intervention prior to their deployment. As the first assessment period started prior to the introduction of the Diagnostic and Statistical Manual of Mental Disorders DSM-5 [23], we added further questions during the post- and follow-up assessment to be able to also investigate PTSD symptoms according to DSM-5. Aiming for phenotypical consistency, we excluded all participants with contradicting PTSD symptom trajectories according to DSM-IV and DSM-5. We divided the remaining 359 soldiers into two groups by subtracting the level of PTSD symptoms of the post-assessment from the level of PTSD symptoms of the follow-up assessment. Individuals with a positive score were assigned to the deteriorating group, whilst individuals with a negative one were assigned to the improving group. Aiming to reduce error variance, we used the MATCH command-line programme created by the Cognitive and Brain Sciences Unit [24] to identify 100 pairs of soldiers consisting of one soldier from the deteriorating and one soldier from the improving group. The soldiers were matched for age, stressful, traumatic and self-perpetrated events prior to the post-assessment, traumatic and violent experiences between the post- and the follow-up assessment, and violence experienced during childhood (see Fig. 1).

### Materials

All instruments were administered as semi‐structured interviews. In addition to the standardised questionnaires, we assessed socio-demographic information.

#### PTSD symptom severity

PTSD symptom severity was assessed using the PTSD Symptom Scale-Interview (PSS‐I) [25]. The PSS-I is a semi-structured interview, which consists of 17 items and has demonstrated validity in comparable East African samples in its self-assessment version [26]. The items correspond to the 17 symptoms of PTSD according to the DSM-IV [27]. DSM-IV was chosen as DSM-5 had not been validated at the time of first data collection. The assessment of symptom severity refers to the last 2 weeks and is based on a 4-point Likert scale ranging from 0 (*not at all*) to 3 (*five or more times per week/almost always*). The total score has a potential range from 0 to 51. In the total sample Cronbach’s α for the scale was 0.88 at post-deployment and 0.93 at follow-up assessment [20, 21].

#### Stressful, traumatic and self-perpetrated events

Exposure to stressful, traumatic and self-perpetrated experiences was assessed using a checklist of 67 event types. The checklist was combined from an adapted checklist used previously in populations affected by violent conflicts including deployment stressors, traumatic events and self-perpetrated violent acts. Binary items were summed to a total score ranging from 0 to 67. Higher values representing a higher exposure to stressful, traumatic, and self-perpetrated events. For more details see Nandi et al. [21]. With this checklist, we assessed lifetime exposure prior to post-deployment and exposure between post- and follow-up assessment.

#### Childhood violence

Exposure to violence during childhood was assessed using a 30-item checklist. The checklist included physical, psychological, and sexual violence as well as neglect and witnessed violence. For each item, participants were asked if they had experienced the event at any time prior to the age of 18. Dichotomous items were summed to a total score ranging from 0 to 30 with higher values indicating more severe childhood violence.

### DNA isolation from human samples

Saliva DNA was collected using an Oragene DNA Kit (DNA Genotek, Ottawa, ONT) and initially extracted using the precipitation protocol recommended by the manufacturer. High-purity DNA was obtained by additional re-purification. For this purpose, 2 µg of DNA isolated via the Oragene procedure was incubated overnight at 50 °C with proteinase K (lysis buffer: 30 mM Tris-HCl pH 8.0, 10 mM EDTA, 1% SDS, 150 ng/l proteinase K), agitated by gentle orbital shaking. Next, the DNA was purified using a Genomic DNA Clean & Concentrator Kit (Zymo Research, Irvine, CA). The DNA quality and concentration were assessed using spectrophotometry (Nanodrop 2000; ThermoScientific, Waltham, MA) and fluorometry (Qubit dsDNA BR Assay Kit, Invitrogen, Carlsbad, CA).

### Infinium EPIC 850 K BeadChip methylation analyses

DNA isolated from the saliva samples was investigated with the Infinium Human Methylation EPIC 850 K array (Illumina, Inc., San Diego, CA). All subjects were processed in a single batch, with a single bisulfite conversion, and with randomised plate assignment that was balanced for all included variables.

During pre-processing, data were extracted and analysed from the generated idat files using the R package RnBeads version 0.99.9 [28]. CpG annotation was based on the manufacturer’s annotation file (Infinium MethylationEPIC v1.0 B5 Manifest File). During pre-processing, the background was subtracted using the “noob” method in the methylumi package [29], and the signal was further normalised using the SWAN algorithm [30]. The following probe categories were excluded from the final data sets, based on the annotation provided within the RnBeads package: non-CpG context probes (due to underrepresentation, 0.6%) [31], functional differences when compared to the CpG context as well as very low abundance of non-CpG methylation in somatic tissues [32] (*N* = 3091); probes with a SNP mapping directly to the target CpG site, as well as probes with three and more SNPs mapping within the 50mer probe (MAF threshold was set to 0.01; *N* = 18,998 CpGs); gonosomal probes (*N* = 11,473 CpGs); non-specific probes. Using the Greedycut algorithm, we iteratively removed the probes and data sets of the highest impurity rows and columns in the detection *p* value table that contain the largest fraction of unreliable measurements; *p* < 0.05; for each sample [28].

The B-values were further post-processed step-by-step in order to correct for further influential and putative confounding factors: (1.) using logit-transformation M-value, [33], done with the R-package car [34]; (2.) z-transformation per plate (correcting for plate and batch effects); (3.) regressing out the first 8 axes of a principal component analysis (PCA, done with the R-package pcaMethods; [35]). The PCA was based on CpGs with no missing values (>95% of the included CpGs). The PCA-based approach corrected for technical biases as well as for part of the variability induced by heterogenous cell composition; (4.) regressing out the effects of sex and age.

The accepted missing rate per CpG was set to <1%. Only samples and CpGs surviving all filtering steps were used for the downstream analyses (*N* = 382 samples – 191 individuals assessed at two time-points; *N* = 817,855 CpG sites).

Finally, we used the genome-wide functional segmentation as specified by the RnBeads package to define promoter and gene-body regions genome-wide [28], and then calculated mean methylation values for each of the elements (GRCh37/hg19; rtracklayer R package [36]). The focus on promoters and gene bodies enabled us to increase power of our analysis, decrease redundancy, and increase the interpretability of our findings given the functional roles of promoters and gene bodies for gene expression regulation.

### Statistical analyses

Statistical analyses were done in SPSS (version 27 [37]), and in R [38]. Given the normal distribution of the DNAm residuals in the human samples, statistical analyses were performed using parametric linear models. All tests were conducted two-sided.

We conducted Welch’s *t* tests to confirm non-significant differences between the groups regarding age, exposure to stressful, traumatic, and self-perpetrated violence, and violence during childhood. The association between DNAm (dependent variable) and PSS-I total scores over time was modelled by using the *cpg.assoc* [39] and *nlme* [40] R packages, with a mixed linear model. We co-variated for the age at t1, the sum of traumatic events until t1, any additional traumatic events between the two assessment points (t1 and t2), and the childhood violence scores (M_value ~ PTSD symptom severity + time + events until t1 + events between t1 and t2 + childhood violence + age + 1/id).

To conduct an exploratory gene-pathway analysis, we selected the nominally significantly associated genes (*P* < 0.05, σ_methyl_ < 0.04; ~700 genes) from the upper model, which were then used for gene enrichment analysis with geneSCF [41] and DAVID [42], using KEGG DB [43] (inclusion criteria: *20* *<* *Pathway Size* < *200; Count* > *2*). Bonferroni single-step process correction was implemented to account for multiple testing procedures. The nominal significance threshold was set in all analyses to *p* = 0.05.

## Results

### Descriptive statistics of the sample

As displayed in Table 1, the 191 male Burundian soldiers were on average 34.45 years old (*SD* = 4.62). They reported on average 24.21 (*SD* = 3.26) traumatic and self-perpetrated violent events prior to or during the AMISOM deployment, and 10.34 (*SD* = 4.30) different types of childhood maltreatment. Confirming the success of the matching procedure the groups with worsening (from 1.9 to 7.0) and improving (7.0 to 1.6) PTSD symptom severity did not differ regarding stressful, traumatic, and self-perpetrated violent events pre- and post-deployment or number of experienced childhood maltreatment events.

### Mixed model analysis

The top nominally significantly associated genes from the mixed model were used for gene enrichment analysis using KEGG DB [43]. Gene set enrichment-like statistical test is applied to determine whether a gene set is enriched for highly ranked *p* values compared to a gene set of identical size, randomly drawn from the genome. We used a gene set size ranging between 20 and 200 genes to avoid both overly narrow and broad functional gene set categories, as well as only gene sets that counted more than 2 genes significantly associated in the mixed model to reduce the bias. This resulted in 49 gene sets to be analysed.

### Gene enrichment analysis

**Fig. 2:**
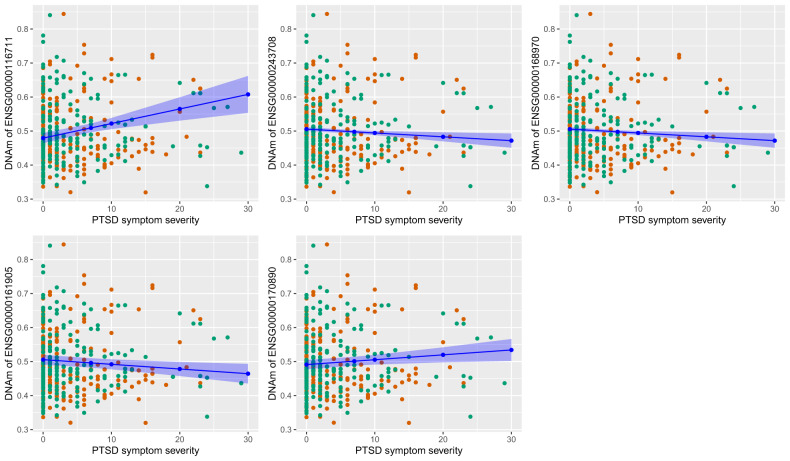
Significant associations of DNA methylation of the 5 identified genes in the linoleic acid metabolism pathway with PTSD symptom severity in male Burundian soldiers after AMISOM deployment. Cross-sectional data from t1 (orange) and t2 (green) are shown. Model estimates are depicted by a blue line, with the corresponding estimate points and confidence intervals also in blue (Estimates of generalised linear models separately for each time point can be found in the Supplementary Fig. 1).

After accounting for multiple comparisons, one KEGG DB pathway remained significant: Linoleic acid metabolism pathway (hsa00591; over enriched 6.97 fold, hypergeometric *P*_nominal_ = 0.0007, *P*_Bonf_ < 0.05). Differential DNAm of 18% of the hsa00591 gene set members (5/28) was significantly associated with the PTSD symptom severity post-deployment, after accounting for covariates as specified by the mixed model (Table 2). Figure 2 displays the methylation of each of these genes in the linoleic acid metabolism pathway versus PTSD symptom severity. Furthermore, the closely related arachidonic and alpha-linoleic pathways were nominally significant but the significance did not survive Bonferroni correction.

**Table 2:** Genes of the linoleic acid metabolism pathway, of which DNAm is significantly associated with PTSD symptom severity.

Gene	T.value	P.value	chr	start	end	symbol
ENSG00000116711	4.1	6.0E-05	chr1	186796585	186798584	PLA2G4A
ENSG00000161905	−2.9	0.004	chr17	4545090	4547089	ALOX15
ENSG00000243708	−2.8	0.006	chr15	42118783	42120782	PLA2G4B
ENSG00000168970	−2.8	0.006	chr15	42118793	42120792	JMJD7-PLA2G4B
ENSG00000170890	2.4	0.017	chr12	120759914	120765592	PLA2G1B

## Discussion

Our study showed that the linoleic acid metabolism KEGG DB pathway was enriched with genes associated with PTSD severity in male Burundian soldiers after AMISOM deployment. Additionally, the associated pathways in alpha-linolenic and arachidonic acid metabolism were nominally associated with PTSD symptom severity. These data indicate that the evolution of PTSD symptoms post-deployment could be associated with substantial epigenetic differences in linoleic acid metabolism and related metabolic pathways.

These acids belong to the family of polyunsaturated fatty acids (PUFA; [44, 45]). While linoleic and arachidonic acids belong to the omega-6 fatty acids, alpha-linolenic acid is an omega-3 fatty acid. Using them as precursors, other omega-6 and omega-3 acids can be manufactured within the body [46]. Previous studies have involved PUFAs in memory related processes and disorders. Linoleic and arachidonic acids levels in brain tissues were found to be associated with Alzheimer’s Disease [47]. A recent study showed that linoleic acid is associated with perceptual speed in non-fasted human subjects [48]. The link between linoleic acid metabolism and memory processes has further been corroborated by a variety of animal studies. A conjugated linoleic acid (CLA) diet in pregnant and lactating rats improved memory outcomes in the offspring [49]. A mixture of alpha-linolenic and linoleic acids administration in rats for 3 weeks was linked to improved learning performance [50]. An experimental study showed that CLA diet also influenced hippocampal mRNA levels and enhanced memory performance in rats [51]. This relationship may be mediated by the buffering effects of PUFA on the stress systems. In line with this hypothesis, an experimental study with students found that intake of a PUFA mixture of omega-3 and −6 improved outcomes linked to test anxiety such as lowering elevated cortisol levels and an associated reduction in anxiety [52]. Furthermore, Yehuda et al. [53] demonstrated that the administration of a mixture of linoleic and alpha-linolenic acids over 3 weeks prevented an increase in cortisol and cholesterol levels in rats after being stressed. The authors also observed a buffering effect for rats treated with linoleic and alpha-linolenic acid for learning deficits. Additionally, PUFA intake seems to protect hippocampal receptors, thus directly affecting neuronal memory structures [52].

In humans, linoleic acid levels at baseline after coronary syndrome have been linked to psychiatric disorder (higher serum linoleic acid level = higher odds ratio (OR) for psychiatric disorder; [54]). In regards to depression symptoms, in perimenopausal women, linoleic acid intake was positively related to depressive symptoms [55]. In persons aged 60 years and above diagnosed with a previous depression in remission, erythrocyte membrane linoleic acid levels revealed an inverted U-shaped curvilinear relationship with depressive and anxiety symptoms. Plasma linoleic acid levels however, showed a negative linear association with depressive symptoms [56]. These results suggest a link between linoleic acid and psychiatric disorders, in particular for depression. This link is in line with our current findings, further indicating that epigenetic regulation may be involved in mediating the relationship between linoleic acid and mental illness.

The relationship between linoleic acid and psychiatric disorders has also been supported in animal studies. In an animal model, CLA supplement in older mice reduced depression-associated signalling in brain to a level of younger mice [57]. Equally important, a high linoleic acid diet before and during pregnancy and during the lactation period in mice was associated with depression-like behaviour in male only offspring [58]. However, the diet did not have an impact on sociability and social recognition memory in male or female subjects.

Arachidonic acid metabolites have been previously linked to cardiovascular diseases, inflammation processes, cancer, arthritis, and metabolic diseases [59, 60]. Furthermore, arachidonic acid is known to promote hippocampal neurogenesis [61]. This could explain, why the authors found arachidonic acid blood levels to be significantly inversely related to risk for PTSD after injury during an accident. Our results associating an elevated DNAm of the gene encoding the enzyme PLA2G4A in individuals with higher PTSD symptom severity levels suggests the same direction. The higher methylation could indicate less PLA2G4A expression and diminished Phospholipase A2 Group IVA activity, subsequently reducing the release of arachidonic acids. Furthermore, the remaining arachnoid acid might quickly be further metabolised by a more active ALOX-15 enzyme.

Studies regarding the importance of omega-3 fatty acids point to the importance of the alpha-linolenic acid metabolism for mental health. Omega-3 deficiency has been linked to a reduction of dopamine vesicle density in the cortex and thus a malfunction of the dopaminergic mesocorticolimbic pathway [46]. Additionally, omega-3 deficiency has been shown to result in a reduction of neuron size in important brain areas such as the hippocampus, hypothalamus, and the cortex, which can eventually lead to learning deficits [62]. Likewise, omega-3 has been linked to immune processes such as induction of a decrease in lymphocyte proliferation, interleukin 1 and 2, and TNF-alpha production [63]. Hence, omega-3 acids seem to decrease the production of pro inflammatory cytokines [46].

Our results hint at the importance of the linoleic acid metabolism pathway, and possibly the very closely related pathways of the arachidonic and alpha-linolenic acid for the evolution of PTSD symptom severity in soldiers in the aftermath of deployment. In line with past epigenetic research these metabolism pathways of PUFAs have been associated with immunological responses, stress reactivity, and memory processes. As PTSD is related to a disconnection of emotional and sensory-related memories from episodic verbally accessible memories [8, 10], the impact of these pathways on hippocampal structures and pathways is of particular significance.

The question remains if those associations must be interpreted cross-sectionally or if some longitudinal causality could be assumed. Even more so, as the reported effects resulted from combining both groups (see Fig. 1). The significant effect of the mixed models resulted mainly from the association between PTSD symptom severity and DNAm. Hence, a conservative interpretation of the data might suggest little evidence of longitudinal causality and assume pre-existing changes in DNAm. However, we need to emphasise that our matched group design with one group ameliorating and one group deteriorating regarding PTSD symptom severity, i.e., every participant having a low and a high value by design, prevented most likely a significant time and/or interaction effect. The strength of the associations between the DNAm of the respective genes and PTSD symptom severity is further corroborated as generalized linear models for each time point showed similar directions of the associations (see Supplementary Fig. 1).

## Limitations and outlook

Our study was able to identify new insights in the deterioration of PTSD symptoms in a Burundian sample, but there are also limiting factors to our study. In general, the level of PTSD symptoms in our sample was below the threshold for a PTSD diagnosis. Although participants were reassured that affirmation of existing PTSD symptoms would not have any negative consequences for them, it cannot be ruled out that participants still did not disclose all of their symptoms. Moreover, we did not control for effects of diet and other lifestyle factors. We can only assume that these factors were less divers than in high income countries, due to relatively standardised living conditions within the military, and some limitations related to poverty. Furthermore, additional biochemical data would have allowed to better understand possible mechanisms and to specify the role of this pathway regarding PTSD symptom development. Nevertheless, our research presents a unique longitudinal study that investigated DNAm patterns linked to PTSD symptom development in a matched group design of active-duty soldiers. As this is the first study associating PTSD symptom severity with the linoleic acid metabolism pathway, future research may further investigate and replicate our results across different samples to explore the generalisability of our findings.

## Conclusion

The present study shows that the linoleic acid metabolism pathway may play a critical role when it comes to the severity of PTSD symptoms in active-duty soldiers. Linoleic acid has previously been linked to memory processes and the immune system in rodents and humans. As memory function constitutes a crucial component of the aetiology of PTSD, our findings suggest a promising new target, the linoleic acid pathway, to better understand the mechanisms for how traumatic experiences interact with biological factors causing PTSD symptoms. This pathway may allow for the influence of memory related aspects of PTSD psychotherapy.

### Supplementary information

Caption Supplementary figure 1

Supplementary figure 1

## Data Availability

Due to the highly sensitive nature of this research, participants of this study did not agree for their data to be made available in a data repository. However, participants gave their informed consent that excerpts of the data that guarantee anonymity can be shared with other researchers upon reasonable request. Please address requests to anselm.crombach@uni-saarland.de.

## Code availability

The R code can be requested from the corresponding author.
