# Supplementary material for: Differential methylation of linoleic acid pathway genes is associated with PTSD symptoms – a longitudinal study with Burundian soldiers returning from a war zone
Source: Transl Psychiatry. 2024 Jan 18;14:32. doi: 10.1038/s41398-024-02757-7 (PMC10796347; doi:10.1038/s41398-024-02757-7)
Supplement: Supplementary file 1 — Caption Supplementary figure 1 [file 41398_2024_2757_MOESM1_ESM.docx]

**Supplementary figure 1**

*Significant associations* *of DNA methylation of the 5 identified genes in the linoleic acid metabolism pathway with PTSD symptom severity in male Burundian soldiers after AMISOM deployment. Cross-sectional data from t1 (orange) and t2 (green) are shown. Generalized linear model estimates are depicted by lines with the corresponding estimate points and confidence intervals in orange and green.*
