# Supplementary figures and images for: Differential methylation of linoleic acid pathway genes is associated with PTSD symptoms – a longitudinal study with Burundian soldiers returning from a war zone
Source: Transl Psychiatry. 2024 Jan 18;14:32. doi: 10.1038/s41398-024-02757-7 (PMC10796347; doi:10.1038/s41398-024-02757-7)

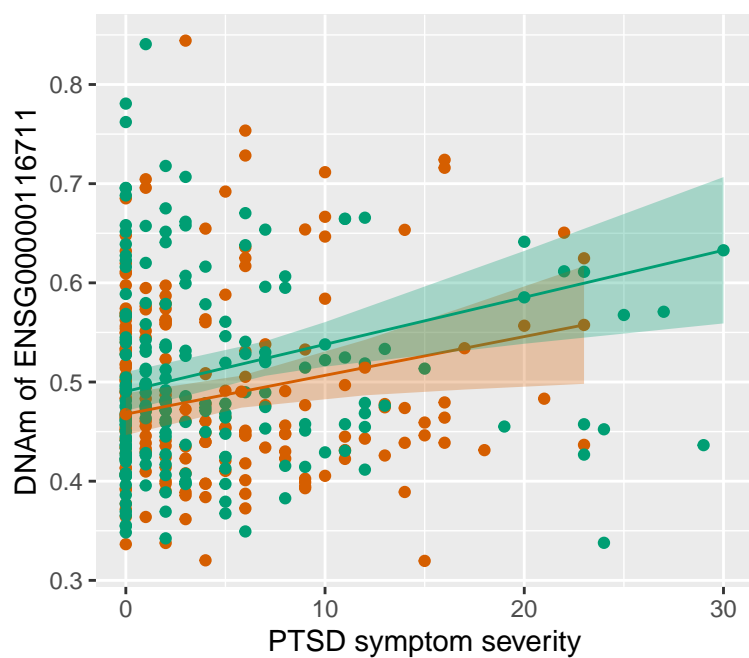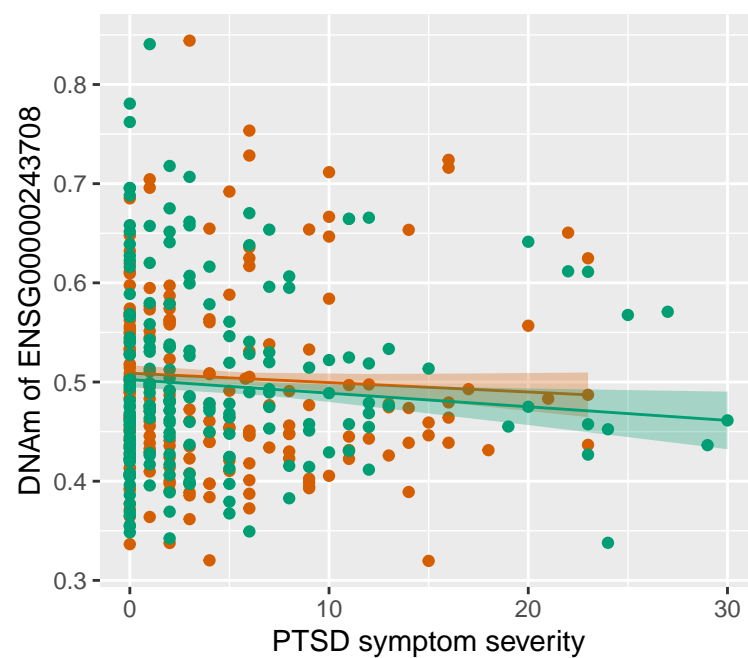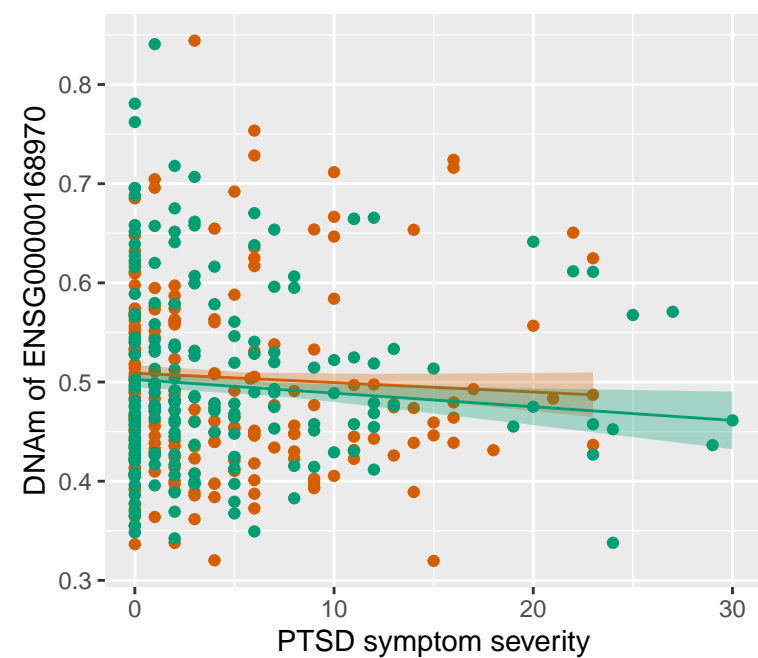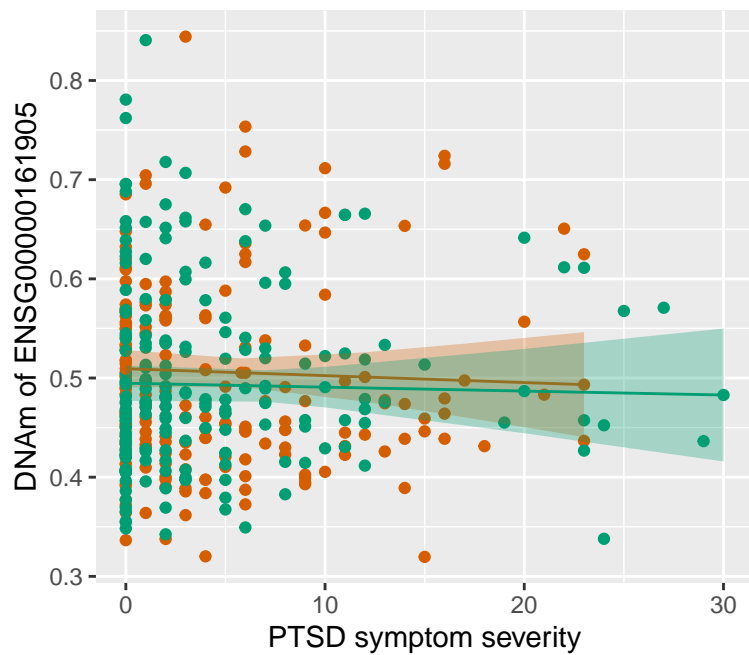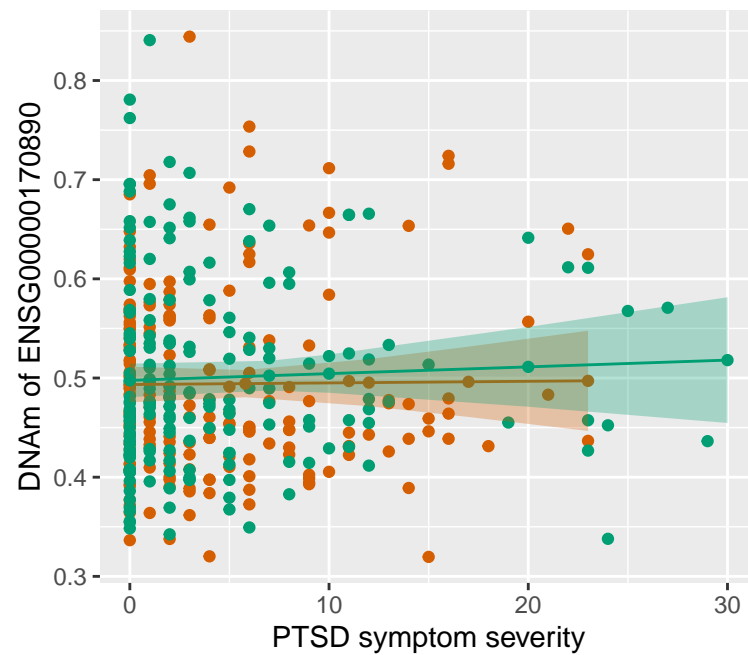

Supplement: Supplementary file 2 — Supplementary figure 1 [file 41398_2024_2757_MOESM2_ESM.pdf]
